# Supplementary material for: In vitro assessment of triterpenoids NVX-207 and betulinyl-bis-sulfamate as a topical treatment for equine skin cancer
Source: PLoS One. 2020 Nov 5;15(11):e0241448. doi: 10.1371/journal.pone.0241448 (PMC7643960; doi:10.1371/journal.pone.0241448)
Supplement: S1 Appendix — IC50 values (μmol/L) of betulinyl-bis-sulfamate (BBS) and NVX-207 thereof on three equine cell types (equine sarcoid [ES] cells sRGO2, equine malignant melanoma [EMM] cells MelDuWi and equine dermal fibroblasts PriFi2) determined by SRB-Assay after 96 h of drug exposure. Measurements were carried out at least as thrice determination. (DOCX) [file pone.0241448.s001.docx]

**S1 Appendix. IC_50_ values measured by SRB Assay after 96 h.** IC_50_ values (µmol/L) of betulinyl-bis-sulfamate (BBS) and NVX-207 thereof on three equine cell types (equine sarcoid [ES] cells sRGO2, equine malignant melanoma [EMM] cells MelDuWi and equine dermal fibroblasts PriFi2) determined by SRB-Assay after 96 h of drug exposure. Measurements were carried out at least as thrice determination.

| Drug | sRGO2 | MelDuWi | PriFri2 |
| --- | --- | --- | --- |
| BBS | 24,34 ± 3,1 | 14,9 ± 1,8 | 19,6 ± 2,2 |
| NVX-207 | 3,1 ± 0,9 | 3,7 ± 0,2 | 3,8 ± 0,4 |
