# Supplementary figures and images for: In vitro assessment of triterpenoids NVX-207 and betulinyl-bis-sulfamate as a topical treatment for equine skin cancer
Source: PLoS One. 2020 Nov 5;15(11):e0241448. doi: 10.1371/journal.pone.0241448 (PMC7643960; doi:10.1371/journal.pone.0241448)

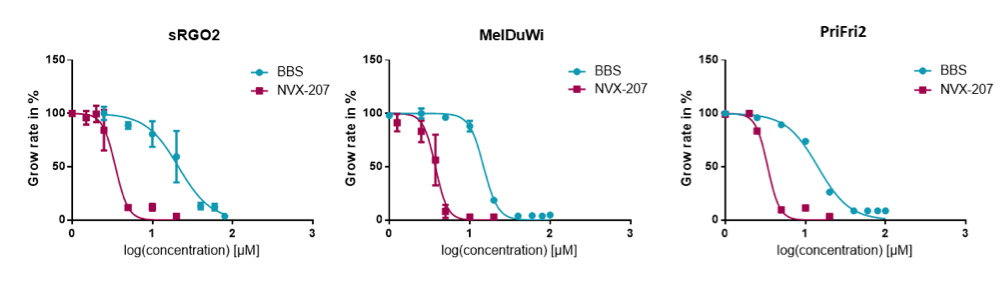

Supplement: S2 Appendix — ES cells sRGO2 (left), EMM (middle) and equine fibroblasts PriFri2 (right) determined by SRB Assay after 96 h (one representative of three independent experiments). (PNG) [file pone.0241448.s002.png]

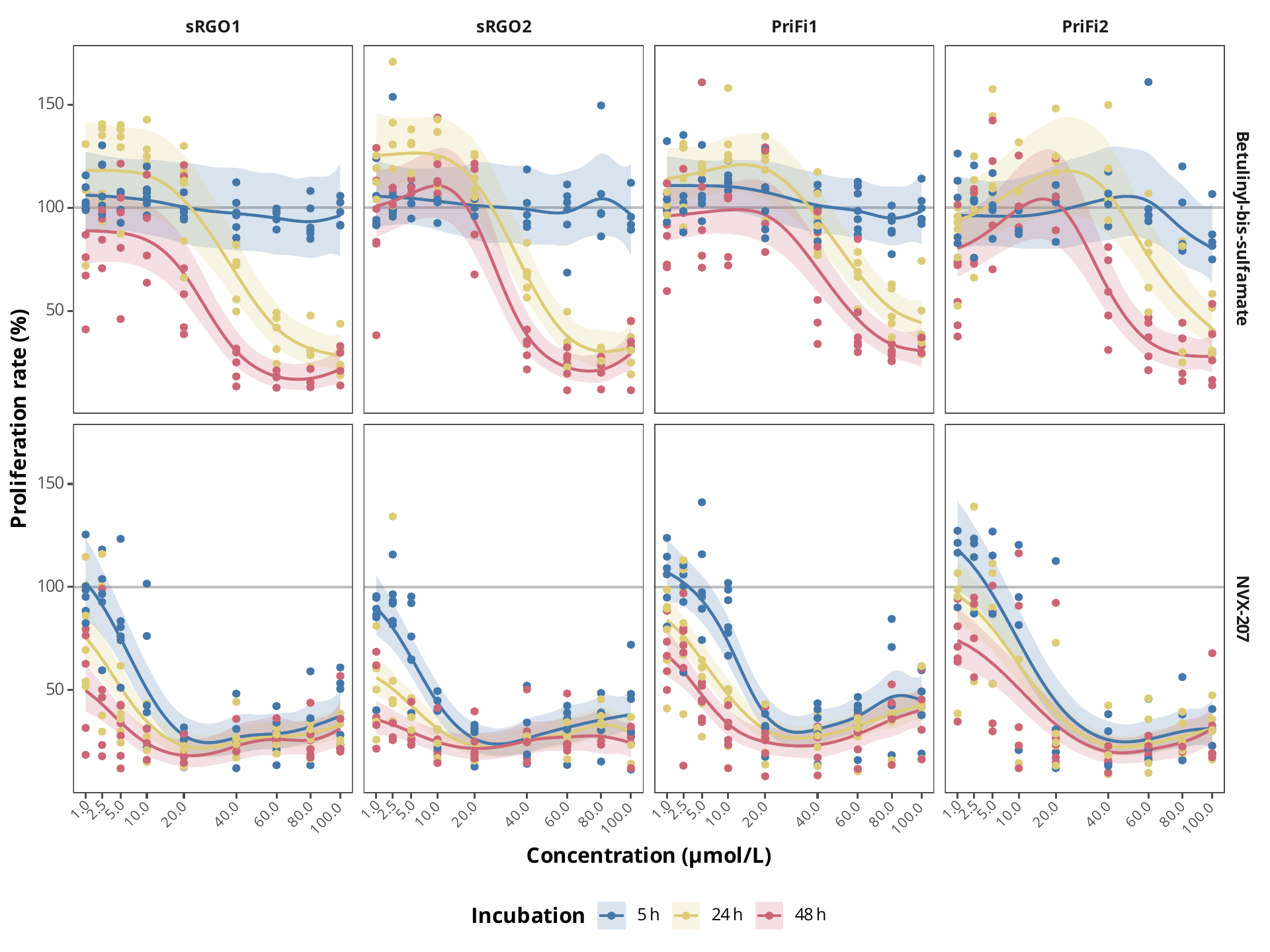

Supplement: S3 Appendix — Proliferation dose-response regression lines of BBS and NVX-207 on primary ES cells (sRGO1, sRGO2) and primary equine dermal fibroblasts (PriFi1, PriFi2) at three different time points (5, 24 and 48 h) determined by crystal violet staining assay. Antiproliferative effects of the compounds on primary equine cells increase with concentration and time of drug exposition. Data represent regression lines and 95% confidence intervals of 6–8 independent experiments for each combination of cell type, incubation time and concentration. Concentrations at which the corresponding 95% confidence intervals do not cross the 100% line indicate a significant reduction of the proliferation rate. (PNG) [file pone.0241448.s003.png]

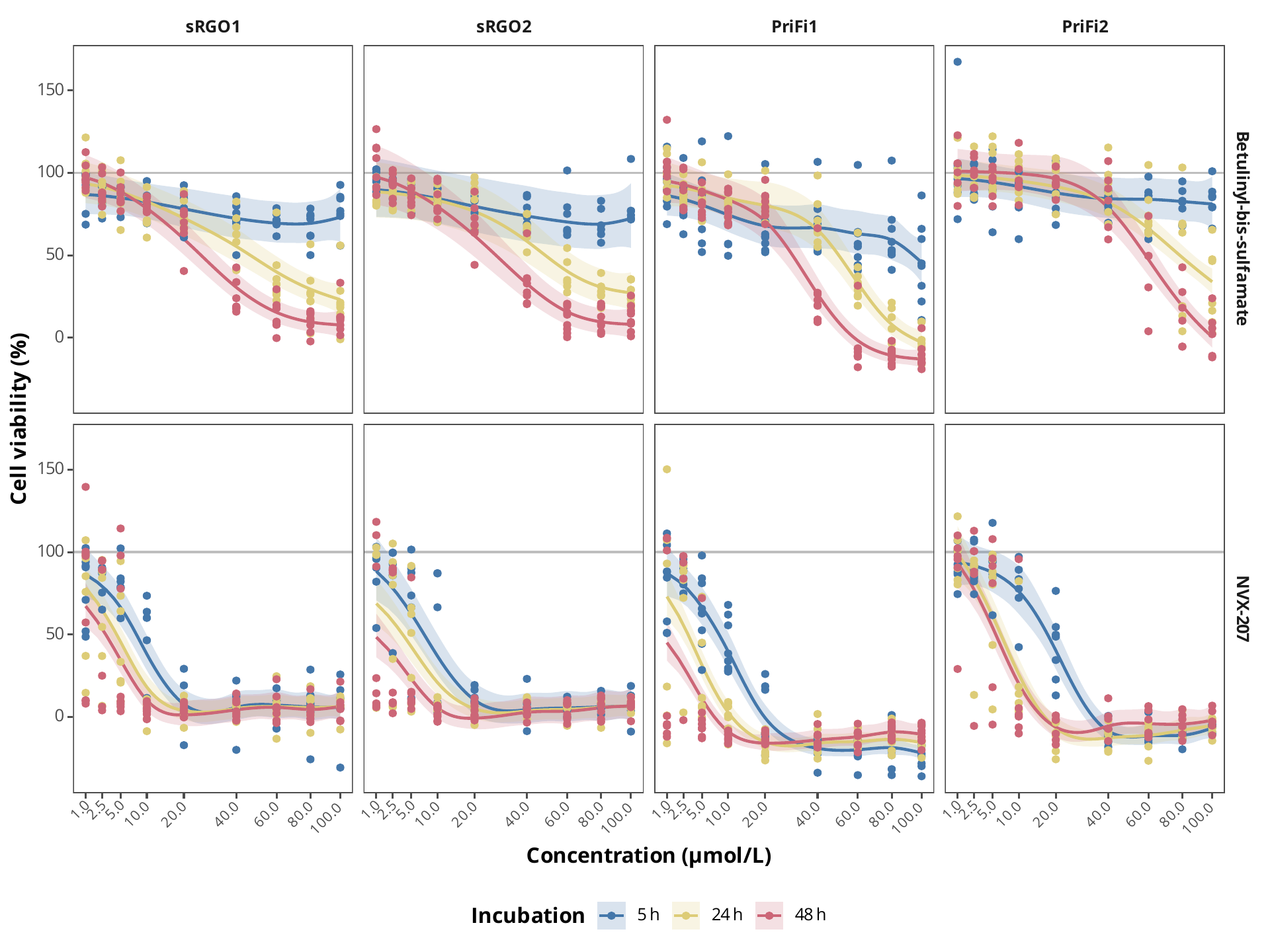

Supplement: S4 Appendix — Proliferation dose-response regression lines of BBS and NVX-207 on primary ES cells (sRGO1, sRGO2) and primary equine dermal fibroblasts (PriFi1, PriFi2) at three different time points (5, 24 and 48 h) determined by MTS assay. Cytotoxic effects of the compounds on primary equine cells increase with concentration and time of drug exposition. Data represent regression lines and 95% confidence intervals of 6–8 independent experiments for each combination of cell type, incubation time and concentration. Concentrations at which the corresponding 95% confidence intervals do not cross the 100% line indicate a significant reduction of the cell viability rate. (PNG) [file pone.0241448.s004.png]

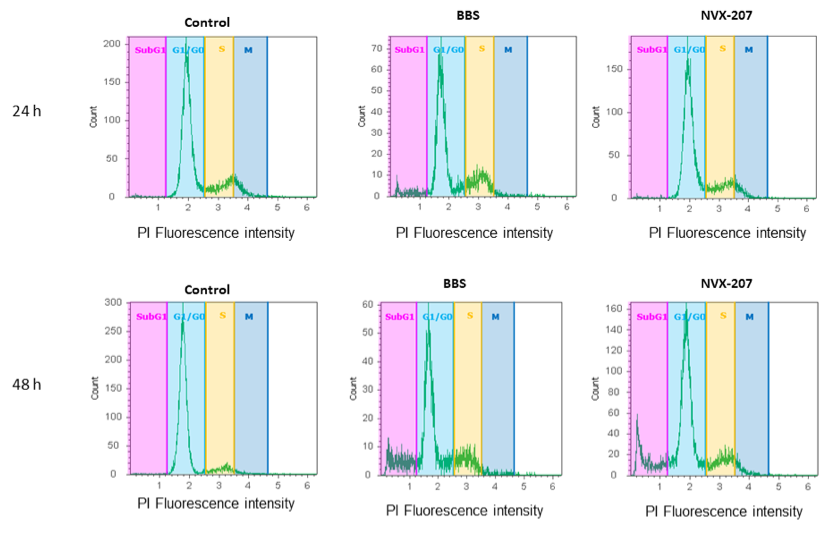

Supplement: S5 Appendix — Cells were untreated (control) or treated with BBS and NVX-207 at their double IC50 concentrations for 24 and 48 h (as indicated). The DNA was stained with propidium iodide and the cells were analyzed by flow cytometry. Red: SubG1 peak; light blue: G1/G0 phase peak; Yellow: S-phase peak; and dark blue: G2/M phase. (PNG) [file pone.0241448.s005.png]

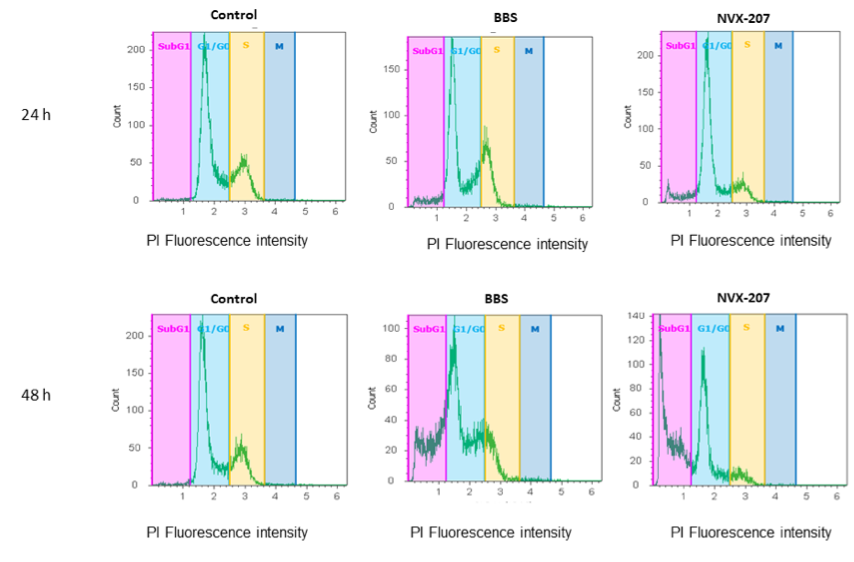

Supplement: S8 Appendix — Cells were untreated (control) or treated with BBS and NVX-207 at their double IC50 concentrations for 24 and 48 h (as indicated). The DNA was stained with propidium iodide and the cells were analyzed by flow cytometry. Red: SubG1 peak; light blue: G1/G0 phase peak; Yellow: S-phase peak; and dark blue: G2/M phase. (PNG) [file pone.0241448.s008.png]

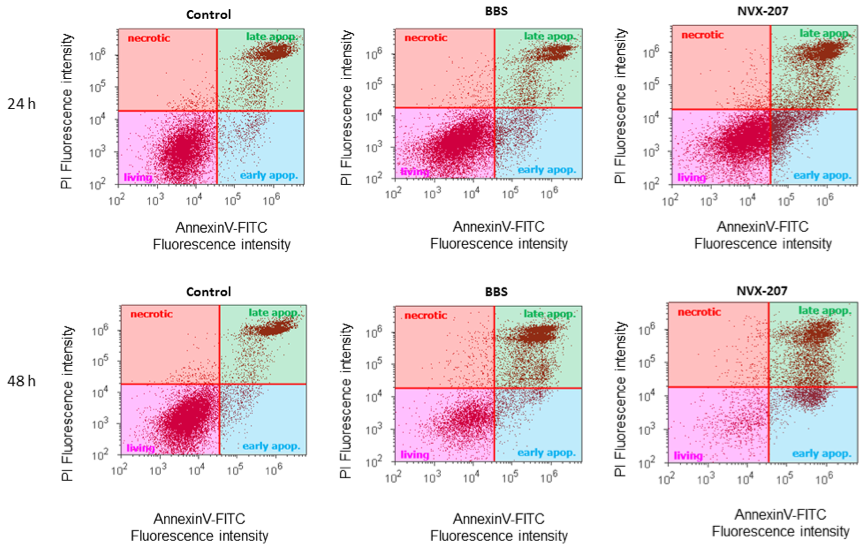

Supplement: S15 Appendix — Cells were untreated (control) or treated with BBS and NVX-207 at their double IC50 concentrations for 24 and 48 h (as indicated). After harvesting, the cells were stained and flow cytometry analysis was performed. Red: necrotic cells; green: late apoptotic cells; blue: early apoptotic cells; magenta: living cells. (PNG) [file pone.0241448.s015.png]

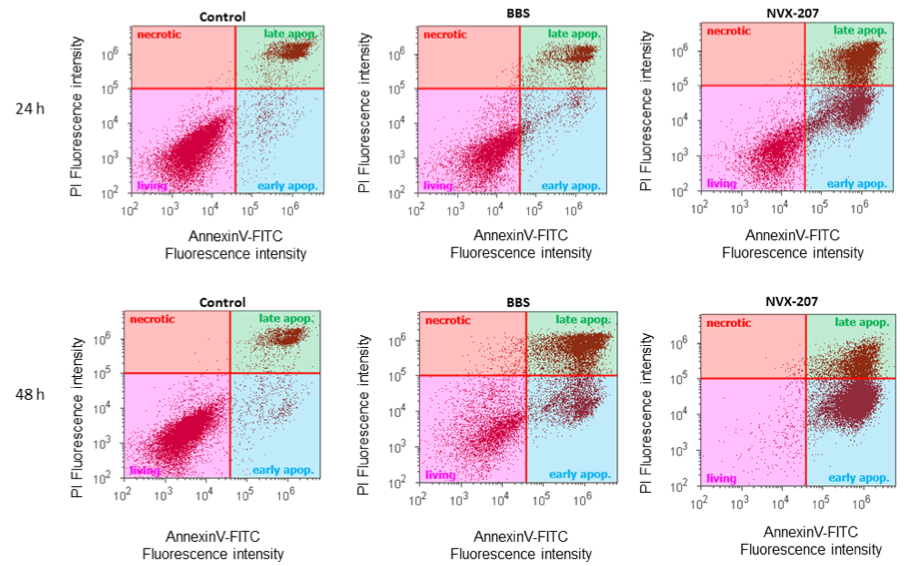

Supplement: S18 Appendix — Cells were untreated (control) or treated with BBS and NVX-207 at their double IC50 concentrations for 24 and 48 h (as indicated). After harvesting, the cells were stained and flow cytometry analysis was performed. Red: necrotic cells; green: late apoptotic cells; blue: early apoptotic cells; magenta: living cells. (PNG) [file pone.0241448.s018.png]

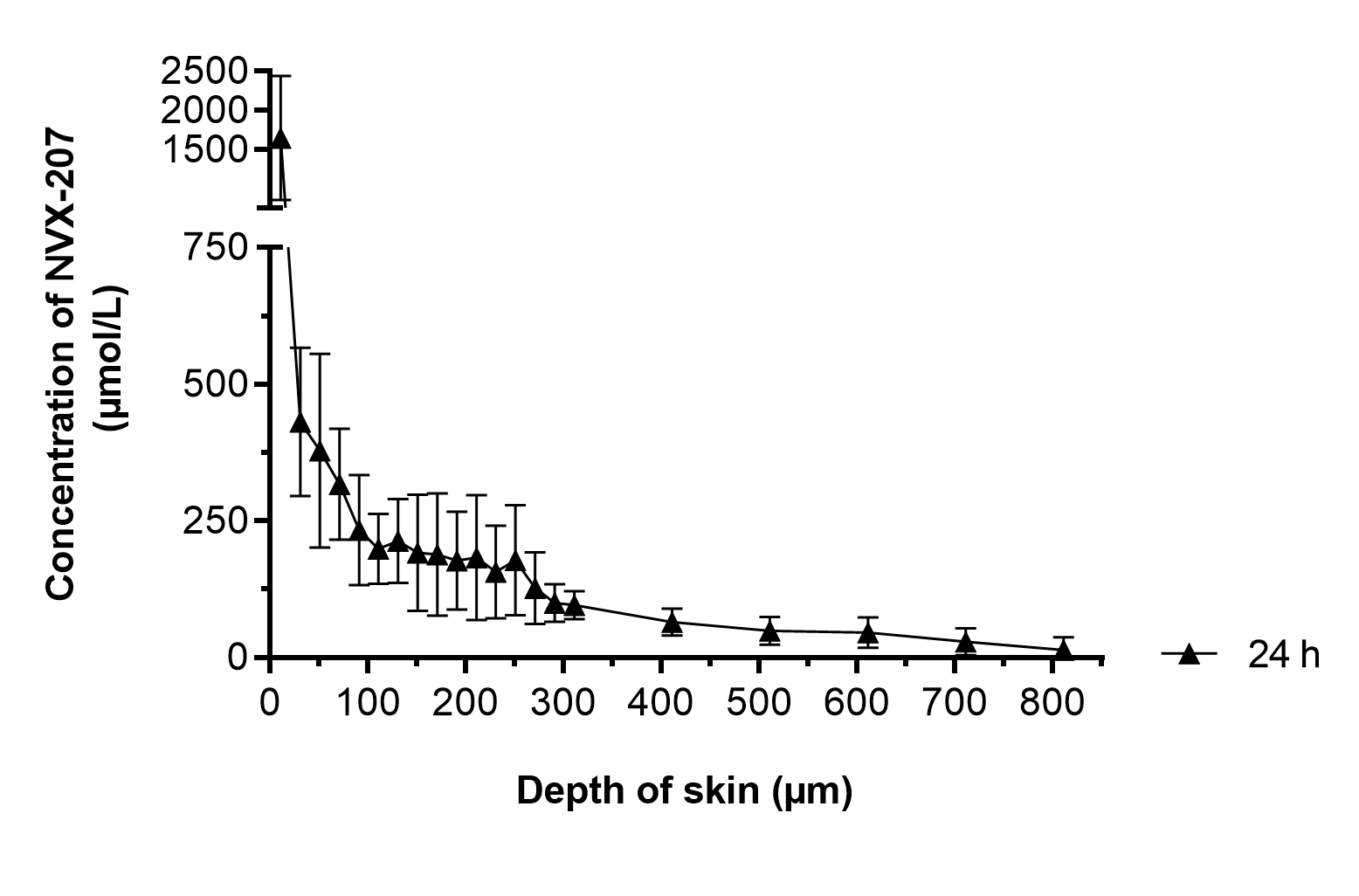

Supplement: S21 Appendix — The skin of six horses (two technical replicates each) were used to investigate the permeation of 1% NVX-207 in “Basiscreme DAC” within 24 h for the Franz-type diffusion cell experiment. The concentration of the compound was determined in 20 μm and 100 μm (deeper skin layers; pooled at 5 × 20 μm) cryostat skin slices at different skin depths by HPLC analysis. Figure data represent mean concentration of NVX-207 at the skin depth indicated and ± SD. Data for 10-μm skin depth (stratum corneum) with potential test compound residues were excluded from this figure. (TIF) [file pone.0241448.s021.tif]
