# Supplementary material for: In vitro assessment of triterpenoids NVX-207 and betulinyl-bis-sulfamate as a topical treatment for equine skin cancer
Source: PLoS One. 2020 Nov 5;15(11):e0241448. doi: 10.1371/journal.pone.0241448 (PMC7643960; doi:10.1371/journal.pone.0241448)
Supplement: S6 Appendix — Cells were untreated (control) or treated with BBS and NVX-207 at their double IC50 concentrations for 24 h. (DOCX) [file pone.0241448.s006.docx]

**S6 Appendix. Cell cycle percentage of ES cells sRGO2.** Cells were untreated (control) or treated with BBS and NVX-207 at their double IC50 concentrations for 24 h.

| 24h | | | |
| --- | --- | --- | --- |
| sRGO2 | Control | BBS | NVX-207 |
| SubG1 | 0,4% | 5,3% | 0,6% |
| G1/G0 | 75,6% | 71,9% | 76,6% |
| S | 15,1% | 21,8% | 15,8% |
| M | 8,4% | 1,6% | 6,4% |
