# Supplementary material for: In vitro assessment of triterpenoids NVX-207 and betulinyl-bis-sulfamate as a topical treatment for equine skin cancer
Source: PLoS One. 2020 Nov 5;15(11):e0241448. doi: 10.1371/journal.pone.0241448 (PMC7643960; doi:10.1371/journal.pone.0241448)
Supplement: S7 Appendix — Cell were untreated (control) or treated with BBS and NVX-207 at their double IC50 concentrations for 48 h. (DOCX) [file pone.0241448.s007.docx]

**S7 Appendix. Cell cycle percentage of ES cells sRGO2.** Cell were untreated (control) or treated with BBS and NVX-207 at their double IC50 concentrations for 48 h.

| 48h | | | |
| --- | --- | --- | --- |
| sRGO2 | Control | BBS | NVX-207 |
| SubG1 | 0,2% | 17,5% | 17,1% |
| G1/G0 | 89,2% | 63,2% | 64,5% |
| S | 8,4% | 16,7% | 13,7% |
| M | 1,9% | 1,8% | 3,9% |
