# Supplementary material for: In vitro assessment of triterpenoids NVX-207 and betulinyl-bis-sulfamate as a topical treatment for equine skin cancer
Source: PLoS One. 2020 Nov 5;15(11):e0241448. doi: 10.1371/journal.pone.0241448 (PMC7643960; doi:10.1371/journal.pone.0241448)
Supplement: S9 Appendix — Cells were untreated (control) or treated with BBS and NVX-207 at their double IC50 concentrations for 24 h. (DOCX) [file pone.0241448.s009.docx]

**S9 Appendix. Cell cycle percentage of EMM MelDuWi.** Cells were untreated (control) or treated with BBS and NVX-207 at their double IC_50_ concentrations for 24 h.

| 24h | | | |
| --- | --- | --- | --- |
| MelDuWi | Control | BBS | NVX-207 |
| SubG1 | 1,1% | 6,7% | 7,1% |
| G1/G0 | 70,0% | 68,2% | 77,4% |
| S | 27,5% | 23,9% | 14,4% |
| M | 0,9% | 0,5% | 0,8% |
