# Supplementary material for: In vitro assessment of triterpenoids NVX-207 and betulinyl-bis-sulfamate as a topical treatment for equine skin cancer
Source: PLoS One. 2020 Nov 5;15(11):e0241448. doi: 10.1371/journal.pone.0241448 (PMC7643960; doi:10.1371/journal.pone.0241448)
Supplement: S10 Appendix — Cells were untreated (control) or treated with BBS and NVX-207 at their double IC50 concentrations for 48 h. (DOCX) [file pone.0241448.s010.docx]

**S10 Appendix. Cell cycle percentage of EMM MelDuWi.** Cells were untreated (control) or treated with BBS and NVX-207 at their double IC_50_ concentrations for 48 h.

| 48h | | | |
| --- | --- | --- | --- |
| MelDuWi | Control | BBS | NVX-207 |
| SubG1 | 1,1% | 43,8% | 60,2% |
| G1/G0 | 70,9% | 48,2% | 26,8% |
| S | 26,8% | 7,6% | 12,9% |
| M | 0,6% | 0,3% | 0,3% |
