# Supplementary material for: In vitro assessment of triterpenoids NVX-207 and betulinyl-bis-sulfamate as a topical treatment for equine skin cancer
Source: PLoS One. 2020 Nov 5;15(11):e0241448. doi: 10.1371/journal.pone.0241448 (PMC7643960; doi:10.1371/journal.pone.0241448)
Supplement: S12 Appendix — Cells were untreated (control) or treated with BBS and NVX-207 at their double IC50 concentrations for 48 h. (DOCX) [file pone.0241448.s012.docx]

**S12** **Appendix. Cell cycle percentage of equine dermal fibroblasts PriFri2.** Cells were untreated (control) or treated with BBS and NVX-207 at their double IC_50_ concentrations for 48 h.

| 48h | | | |
| --- | --- | --- | --- |
| PriFri2 | Control | BBS | NVX-207 |
| SubG1 | 1,5% | 13,7% | 81,9% |
| G1/G0 | 80,1% | 73,7% | 14,8% |
| S | 15,9% | 10,8% | 2,9% |
| M | 1,6% | 0,9% | 0,1% |
