# Supplementary material for: In vitro assessment of triterpenoids NVX-207 and betulinyl-bis-sulfamate as a topical treatment for equine skin cancer
Source: PLoS One. 2020 Nov 5;15(11):e0241448. doi: 10.1371/journal.pone.0241448 (PMC7643960; doi:10.1371/journal.pone.0241448)
Supplement: S13 Appendix — Percentage of ES cells sRGO2 untreated (control) or treated with BBS and NVX-207 at their double IC50 concentrations for 24 h. (DOCX) [file pone.0241448.s013.docx]

**S13 Appendix. AnnexinV staining.** Percentage of ES cells sRGO2 untreated (control) or treated with BBS and NVX-207 at their double IC_50_ concentrations for 24 h.

| 24h | | | |
| --- | --- | --- | --- |
| sRGO2 | control | BBS | NVX-207 |
| Living cells | 83,4% | 41,1% | 76,6% |
| early apop | 1,8% | 19,1% | 3,7% |
| late apop | 14,3% | 38,5% | 19,1% |
| necrotic | 0,5% | 1,3% | 0,5% |
