# Supplementary material for: In vitro assessment of triterpenoids NVX-207 and betulinyl-bis-sulfamate as a topical treatment for equine skin cancer
Source: PLoS One. 2020 Nov 5;15(11):e0241448. doi: 10.1371/journal.pone.0241448 (PMC7643960; doi:10.1371/journal.pone.0241448)
Supplement: S14 Appendix — Percentage of ES cells sRGO2 untreated (control) or treated with BBS and NVX-207 at their double IC50 concentrations for 48 h. (DOCX) [file pone.0241448.s014.docx]

**S14 Appendix. AnnexinV staining.** Percentage of ES cells sRGO2 untreated (control) or treated with BBS and NVX-207 at their double IC_50_ concentrations for 48 h.

| 48h | | | |
| --- | --- | --- | --- |
| sRGO2 | control | BBS | NVX-207 |
| Living cells | 79,7% | 8,7% | 38,5% |
| early apop | 2,4% | 7,5% | 12,5% |
| late apop | 17,3% | 82,1% | 47,9% |
| necrotic | 0,6% | 1,8% | 1,1% |
