# Supplementary material for: In vitro assessment of triterpenoids NVX-207 and betulinyl-bis-sulfamate as a topical treatment for equine skin cancer
Source: PLoS One. 2020 Nov 5;15(11):e0241448. doi: 10.1371/journal.pone.0241448 (PMC7643960; doi:10.1371/journal.pone.0241448)
Supplement: S17 Appendix — Percentage of equine dermal fibroblasts PriFri2 untreated (control) or treated with BBS and NVX-207 at their double IC50 concentrations for 48 h. (DOCX) [file pone.0241448.s017.docx]

**S17 Appendix. AnnexinV staining.** Percentage of equine dermal fibroblasts PriFri2 untreated (control) or treated with BBS and NVX-207 at their double IC_50_ concentrations for 48 h.

| 48h | | | |
| --- | --- | --- | --- |
| PriFri2 | control | BBS | NVX-207 |
| Living cells | 77,4% | 33,1% | 12,3% |
| early apop | 3,0% | 9,3% | 17,9% |
| late apop | 18,6% | 53,6% | 67,1% |
| necrotic | 1,2% | 4,1% | 1,8% |
