# Supplementary material for: In vitro assessment of triterpenoids NVX-207 and betulinyl-bis-sulfamate as a topical treatment for equine skin cancer
Source: PLoS One. 2020 Nov 5;15(11):e0241448. doi: 10.1371/journal.pone.0241448 (PMC7643960; doi:10.1371/journal.pone.0241448)
Supplement: S20 Appendix — Percentage of EMM cells (MelDuWi) untreated (control) or treated with BBS and NVX-207 at their double IC50 concentrations for 48 h. (DOCX) [file pone.0241448.s020.docx]

**S20 Appendix*.* AnnexinV staining.** Percentage of EMM cells (MelDuWi) untreated (control) or treated with BBS and NVX-207 at their double IC_50_ concentrations for 48 h.

| 48h | | | |
| --- | --- | --- | --- |
| MelDuWi | control | BBS | NVX-207 |
| Living cells | 82,2% | 60,6% | 29,8% |
| early apop | 2,3% | 7,5% | 24,3% |
| late apop | 15,0% | 28,2% | 44,8% |
| necrotic | 0,5% | 3,7% | 1,0% |
