# Supplementary material for: In vitro assessment of triterpenoids NVX-207 and betulinyl-bis-sulfamate as a topical treatment for equine skin cancer
Source: PLoS One. 2020 Nov 5;15(11):e0241448. doi: 10.1371/journal.pone.0241448 (PMC7643960; doi:10.1371/journal.pone.0241448)
Supplement: Attachment — Submitted filename: Response to Reviewers.docx [file pone.0241448.s022.docx]

Hannover, 29^th^ of September 2020

**Subject: Submission of revised paper PONE-D-20-12654 entitled *“In vitro assessment of triterpenoids NVX-207 and betulinyl-bis-sulfamate as a topical treatment for equine skin cancer”***

Dear Dr. Lebedeva and reviewers,

the authors would like to thank the editor and reviewers for the review of the manuscript PONE-D-20-12654 entitled "In vitro assessment of triterpenoids NVX-207 and betulinyl-bis-sulfamate as a topical treatment for equine skin cancer". We have carefully considered your comments and our responses are given in a point-by-point manner below. Line numbers included in the responses refer to the updated version of the manuscript.

2.1. Please provide an amended Funding Statement declaring this commercial affiliation, as well as a statement regarding the Role of Funders in your study. If the funding organization did not play a role in the study design, data collection and analysis, decision to publish, or preparation of the manuscript and only provided financial support in the form of authors' salaries and/or research materials, please review your statements relating to the author contributions, and ensure you have specifically and accurately indicated the role(s) that these authors had in your study. You can update author roles in the Author Contributions section of the online submission form.

Dr. Jutta Kalbitz and Prof. Dr. Reinhard Paschke were funded by BioSolutions Halle GmbH with project funds provided by the German Federal Ministry for Economic Affairs and Engery. The TopiDrugHorse project is a cooperation project between research institutions and a company. The participation of a company in this cooperation project is the prerequisite for the approval of the project funds by the Ministry of Economic Affairs and Energy. BioSolutions Halle GmbH is therefore not a commercial funder, but an equal partner. The rights and obligations of all partners are governed by a cooperation agreement. This applies in particular to publications and intellectual property. We would be grateful if you could give us feedback on whether this modified funding statement is sufficient or whether you need further information:

*"Some authors were funded by the Central Innovation Programme of the German Federal Ministry for Economic Affairs and Energy. LAW: Specific grant number: TopiDrugHorse 16KN051526 BMWI; AF: Specific grant number: TopiDrugHorse 16KN051530 BMWI; RP: Specific grant number: TopiDrugHorse 16KN051524 BMWI; JK: Specific grant number: TopiDrugHorse 16KN051524 BMWI. RP and JK were further funded by BioSolutions Halle GmbH (www.biosolutions-halle.de). The TopiDrugHorse project is a cooperation project between research institutions and a company. The participation of a company in this cooperation project is the prerequisite for the approval of the project funds by the Ministry of Economic Affairs and Energy. BioSolutions Halle GmbH is therefore not a commercial funder, but an equal partner. The rights and obligations of all partners are governed by a cooperation agreement. This applies in particular to publications and intellectual property. The funders provided support in the form of salaries and/or research materials for authors [LAW, AF, RP, JK], but did not have any additional role in the study design, data collection and analysis, decision to publish, or preparation of the manuscript. The specific roles of these authors are articulated in the ‘author contributions’ section.”*

2.2. Please also provide an updated Competing Interests Statement declaring this commercial affiliation along with any other relevant declarations relating to employment, consultancy, patents, products in development, or marketed products, etc. Within your Competing Interests Statement, please confirm that this commercial affiliation does not alter your adherence to all PLOS ONE policies on sharing data and materials by including the following statement: "This does not alter our adherence to PLOS ONE policies on sharing data and materials.”

The affiliation “BioSolutions Halle GmbH” of Dr. Jutta Kalbitz does not alter our adherence to PLOS ONE policies on sharing data and materials. As described in the Funding Statement, BioSolutions Halle GmbH is not a commercial funder, but an equal partner in the TopiDrugHorse project. Therefore, we are of the opinion that the conflict of interest statement does not need to be updated.

*“The authors have declared that no competing interests exist.”*

If the sentence you proposed must be included, we edit the conflict of interest statement as follows:

*“The authors declare that no competing interests exist. The affiliation “BioSolutions Halle GmbH” of Dr. Jutta Kalbitz does not alter our adherence to PLOS ONE policies on sharing data and materials. As described in the Funding Statement, BioSolutions Halle GmbH is not a commercial funder, but an equal partner in the TopiDrugHorse project.”*

3. We note that you have included the phrase “data not shown” in your manuscript. Unfortunately, this does not meet our data sharing requirements. PLOS does not permit references to inaccessible data. We require that authors provide all relevant data within the paper, Supporting Information files, or in an acceptable, public repository. Please add a citation to support this phrase or upload the data that corresponds with these findings to a stable repository (such as Figshare or Dryad) and provide and URLs, DOIs, or accession numbers that may be used to access these data. Or, if the data are not a core part of the research being presented in your study, we ask that you remove the phrase that refers to these data.

Thank you for the comment. We removed the phrase “data not shown” in the revised version of the manuscript as the data are not a core part of the research being presented in the study.

**Comments to the Author**

Reviewer #1: In this manuscript, the authors have assessed the topical application of triterpenoids NVX-207 and betulinyl-bis-sulfamate as a topical treatment for equine skin cancer. This is a follow-up in vivo study of their in vitro work published earlier.

The assays proposed in the manuscript are standard for use for such studies. The main issue is the absence of normal melanocytes as a control for the experiments with melanoma cells. The use of fibroblasts is not an ideal control for these studies.

We thank reviewer #1 for the review of our manuscript. We would like to point out that a safety study on the target animal (horse) has been published recently by us [1]. It is likely that this publication was not available to reviewer #1 at the time the current report was prepared. Within the frame of the safety study the skin of eight healthy horses were treated percutaneously in a crossover design with 1% betulinic acid, 1% NVX-207 or a placebo in a respective vehicle twice a day for seven consecutive days. In the histopathological examination of the treated skin no compound-related local adverse effects were observed. Therefore, we are of the opinion that a series of experiments with normal equine melanocytes does not lead to further valuable insights in the development of a topical drug against equine skin cancer. Still, we have conducted an extensive literature research to find publications on the *in vitro* culture and treatment of normal equine melanocytes. Please note that to the best of our knowledge no data exist for *in vitro* cell culture experiments with this cell type. We hope that reviewer #1 will agree with us that the establishment of a protocol to generate and cultivate an equine melanocyte cell line would go beyond the scope of the current manuscript. We agree with reviewer #1 that equine dermal fibroblasts are not an ideal control for equine melanoma cells. However, fibroblasts are often used to gain insights into anticancer drugs’ cytotoxicity to normal cells and to investigate anticancer drugs’ *in vitro* selectivity indices [2–6]. Besides, dermal fibroblasts provide an ideal control to equine sarcoid cells as equine sarcoids are tumors of fibroblasts [7]. In order to address the current *in vivo* data regarding the safety of NVX-207 in horses we included the following sentences in the introduction and discussion sections:

*“In addition, the compound was already successfully tested in a clinical study with canine cancer patients [64]. Within the frame of pilot safety studies, NVX-207 was well tolerated when applied topically in eight healthy horses [67] or injected intralesionally in two horses affected by EMM [66].“* **(lines 115-118)**

*“Furthermore, current in vivo data indicate a good systemic and local tolerability of 1 % NVX-207 after topical application in eight healthy horses twice a day for seven consecutive days [67]. In addition, the intralesional injection of the compound in two EMM patients once a week for 19 consecutive weeks proveed to be safe [66]. (…)”* **(lines 470-474)**

Another concern is the resolution of figures, which makes them hard to read.

Thank you for the note*.* We provided high resolution figures in the revised manuscript.

Reviewer #2: Is the manuscript presented in an intelligible fashion and written in standard English? Reviewer #2: No

We thank reviewer #2 for the hint. Please note that the resubmitted manuscript has been professionally edited for English language by an English language support service (Philip Saunders; http://www.saundersproof.de/en/en.html).

This study is a follow-on study that offers further details on the possible antitumor effects of the triterpenoids NVX-207 and 3 betulinyl-bis-sulfamate on a variety of equine tumor cells. Although the findings are interesting, there are some limitations to this study:
First, it would have been helpful to add a positive control to the cell assays (i.e. an anti-tumor drug for which thorough data on cytotoxicity and cell proliferation are available.) E.g. comparison to imiquimod would be interesting, as this is also used for treatment of equine sarcoids. The general limitation is, that the MTS assay displays a cytotoxic effect for both NVX-207 and 3 betulinyl-bis-sulfamate and the index reduction of proliferation/reduction of cell viability does not look convincing for either of the substances. In addition, the cytotoxic effect on normal fibroblasts might limit its use further.
These data will only add fundamentally new insights when combined with repeated local tolerance tests (e.g. topical administration of a NVX-207 to mouse skin over a time period intended for in-vivo use in horses).

We thank reviewer #2 for the review of our manuscript. We disagree that the absence of positive controls is a limitation of the study. We have waived the positive control in the cell culture experiments as there exist no evidence-based *in vitro* data on cytotoxicity, cell proliferation, or detailed molecular pathways for anti-tumor drugs and equine cancer cells that could have been applied for our experiments and would bring any further gain in knowledge. Indeed, the immune system modifier imiquimod is used for equine sarcoid treatment; however only data for *in vivo* use in horses are available [9,10]. We agree that a reliable transferability of *in vitro* results gained from cell culture experiments to *in vivo* conditions is not always given and *in vivo* studies should follow the initial *in vitro* investigations. We conducted an *in vivo* safety study in eight healthy horses which was recently published [1] and which reviewer #2 was probably not yet available at the time of writing the current report. Good systemic tolerability and only mild local vehicle-related but not compound-related adverse effects were observed after topical application of 1 % betulinic acid and 1 % NVX-207, respectively. The histopathological examination revealed no cytotoxic effects of the compounds in normal dermal fibroblasts. To address the recently published *in vivo* data we included the following sentences in the introduction and discussion sections.

*“In addition, the compound was already successfully tested in a clinical study with canine cancer patients [64]. Within the frame of pilot safety studies, NVX-207 was well tolerated when applied topically in eight healthy horses [67] or injected intralesionally in two horses affected by EMM [66].“* **(lines 115-118)**

*“Furthermore, current in vivo data indicate a good systemic and local tolerability of 1 % NVX-207 after topical application in eight healthy horses twice a day for seven consecutive days [67]. In addition, the intralesional injection of the compound in two EMM patients once a week for 19 consecutive weeks proved to be safe [66]. (…)”* **(lines 470-474)**

To emphasize that results obtained from this *in vitro* study are not necessarily applicable to the clinical situation we included the following sentences in the discussion:

*“Even though the results reported from in vitro experiments with equine skin cancer cells are promising, it must be emphasized that cells in their native microenvironment can be much more robust against (phyto)chemotherapeutic influences [74]–[76]. Therefore, no reliable conclusions can be drawn regarding the efficacy of a topical NVX-207 application in ES and EMM patients and prospective in vivo studies have made to address this question.” (***lines 504-509)**

Regarding the fact that *in vivo* safety studies have already been conducted and published, the conclusion of the original version of the manuscript is not actual anymore (“As a cytotoxicity to normal dermal fibroblasts has been observed as well, in vivo safety studies on normal equine skin should be performed beforehand.“). Therefore, we have revised the conclusion in the abstract and discussion, which now reads as follows:

*“Even though data obtained in vitro are auspicious, the results are not unconditionally applicable to the clinical situation. Consequently, in vivo studies are required to address the antitumoral effects of topically applied NVX-207 in ES and EMM patients.”* **(lines 45-47)**

*“Although the in vitro data reported are promising, the results are not unconditionally applicable to the clinical situation. Therefore, in vivo studies are needed to assess the antitumoral effects of topically applied NVX-207 in equine patients suffering from ES or EMM.”* **(lines 604-607)**

We thank you kindly for your contributions to this manuscript and we look forward to your response.

Yours sincerely,

Jessika-M.V. Cavalleri	Lisa A. Weber

**REFERENCES**

[1]	Weber LA, Puff C, Kalbitz J, Kietzmann M, Feige K, Bosse K, et al. Concentration profiles and safety of topically applied betulinic acid and NVX‐207 in eight healthy horses—A randomized, blinded, placebo‐controlled, crossover pilot study. J Vet Pharmacol Ther 2020;0:jvp.12903. https://doi.org/10.1111/jvp.12903.

[2]	Willmann M, Wacheck V, Buckley J, Nagy K, Thalhammer J, Paschke R, et al. Characterization of NVX-207, a novel betulinic acid-derived anti-cancer compound. Eur J Clin Invest 2009;39:384–94. https://doi.org/10.1111/j.1365-2362.2009.02105.x.

[3]	Mullauer FB, Kessler JH, Medema JP. Betulinic acid induces cytochrome c release and apoptosis in a Bax/Bak-independent, permeability transition pore dependent fashion. Apoptosis 2009;14:191–202. https://doi.org/10.1007/s10495-008-0290-x.

[4]	Zuco V, Supino R, Righetti SC, Cleris L, Marchesi E, Gambacorti-Passerini C, et al. Selective cytotoxicity of betulinic acid on tumor cell lines, but not on normal cells. Cancer Lett 2002;175:17–25. https://doi.org/10.1016/S0304-3835(01)00718-2.

[5]	Baratto LC, Porsani M V., Pimentel IC, Pereira Netto AB, Paschke R, Oliveira BH. Preparation of betulinic acid derivatives by chemical and biotransformation methods and determination of cytotoxicity against selected cancer cell lines. Eur J Med Chem 2013;68:121–31. https://doi.org/10.1016/j.ejmech.2013.07.012.

[6]	Kommera H, Kaluderović GN, Dittrich S, Kalbitz J, Dräger B, Mueller T, et al. Carbamate derivatives of betulinic acid and betulin with selective cytotoxic activity. Bioorganic Med Chem Lett 2010;20:3409–12. https://doi.org/10.1016/j.bmcl.2010.04.004.

[7]	Knottenbelt DC. A suggested clinical classification for the equine sarcoid. Clin Tech Equine Pract 2005;4:278–95. https://doi.org/10.1053/j.ctep.2005.10.008.

[8]	Liebscher G, Vanchangiri K, Mueller T, Feige K, Cavalleri JMV, Paschke R. In vitro anticancer activity of Betulinic acid and derivatives thereof on equine melanoma cell lines from grey horses and invivo safety assessment of the compound NVX-207 in two horses. Chem Biol Interact 2016;246:20–9. https://doi.org/10.1016/j.cbi.2016.01.002.

[9]	Nogueira SAF, Torres SMF, Malone ED, Diaz SF, Jessen C, Gilbert S. Efficacy of imiquimod 5% cream in the treatment of equine sarcoids: A pilot study. Vet Dermatol 2006;17:259–65. https://doi.org/10.1111/j.1365-3164.2006.00526.x.

[10]	Haspeslagh M, Vlaminck LEM, Martens AM. Treatment of sarcoids in equids: 230 cases (2008–2013). J Am Vet Med Assoc 2016;249:311–8. https://doi.org/10.2460/javma.249.3.311.
